# High expression levels of class III β-tubulin in resected non-small cell lung cancer patients are predictive of improved patient survival after vinorelbine-based adjuvant chemotherapy

**DOI:** 10.3892/ol.2013.1323

**Published:** 2013-04-29

**Authors:** YALEI ZHANG, HAIHONG YANG, JUN LIU, QIUHUA DENG, PING HE, YUNEN LIN, JUHONG JIANG, XIA GU, MINGCONG MO, HUI PAN, XINGUO XIONG, YUAN QIU, JIANXING HE

**Affiliations:** 1 Southern Medical University, Guangzhou, Guandong 510515;; 2 Department of Cardiothoracic Surgery, The First Affiliated Hospital of Guangzhou Medical College, Guangzhou, Guandong 510120;; 3 Department of Cardiothoracic Surgery, Nanfang Hospital, Southern Medical University, Guangzhou, Guandong 510515;; 4 Department of Pathology, The First Affiliated Hospital of Guangzhou Medical College, Guangzhou, Guandong 510120, P.R. China

**Keywords:** class III β-tubulin, resected non-small cell lung cancer, adjuvant chemotherapy

## Abstract

The aim of the present study was to determine the frequency and predictive value of the expression of tumor microtubule components in patients with resected non-small cell lung cancer (R-NSCLC) subsequently treated with vinorelbine-based adjuvant chemotherapy. The expression of the microtubule components was evaluated in 85 R-NSCLC tumor samples using immunohistochemistry. All patients received vinorelbine-based chemotherapy. The predictive value of microtubule protein expression for disease-free survival (DFS) and overall survival (OS) was assessed. The expression of the microtubule components was not associated with any baseline clinicopathological factors in the R-NSCLC patients. High tumor expression levels of class III β-tubulin were correlated with an improved DFS (P=0.033) and a trend towards a longer OS (P=0.226). Class II and IV β-tubulins were not correlated with patient outcome. Multivariate analysis of factors, including gender, age, histology, stage and class II, III and IV β-tubulin expression demonstrated that high levels of class III β-tubulin expression were correlated independently with DFS (P= 0.031). These findings suggest that high class III β-tubulin expression levels in resected tumors are predictive of improved DFS in R-NSCLC patients receiving vinorelbine-based chemotherapy.

## Introduction

In 2008, lung cancer was the most commonly diagnosed cancer, accounting for 1.6 million new cancer cases and 13% of all new cancer cases. Lung cancer was also the leading cause of cancer-related mortality in males worldwide, responsible for 1.4 million mortalities, which corresponded to 18% of all cancer-related mortalities in 2008 (1). Surgical resection remains the treatment of choice for stage I and II non-small cell lung cancer (NSCLC) and also for certain patients with stage IIIA disease. A number of studies have shown that platinum-based adjuvant chemotherapy improves survival in completely resected NSCLC (R-NSCLC) (2–4).

Although adjuvant chemotherapy provides a clinically significant benefit, only between 5 and 15% of NSCLC patients receiving adjuvant chemotherapy ultimately benefit from an improved long-term survival (5). Given the toxic effects of chemotherapy and the resources required to administer this treatment, it would be useful to identify which NSCLC patients are likely to benefit from adjuvant chemotherapy prior to treatment. The optimization of current therapeutic strategies would greatly benefit from the identification of novel predictive factors, taking into account the patient’s genetics and the biological characteristics of their disease. Such predictive factors may be used to effectively guide the clinician’s decisions.

Antitubulin agents, including taxanes and vinorelbine, bind to microtubules and are widely used for the treatment of NSCLC. Microtubules are dynamic filamentous structures that are essential in eukaryotic cell proliferation, intracellular trafficking, signaling and migration. Microtubules are complex polymers consisting of tubulin dimers, each containing one α-tubulin and one β-tubulin molecule, and a variety of microtubule-associated proteins (6). In humans, β-tubulin exists as a variety of isotypes that mainly differ in their COOH-terminal sequences (7).

Little data is available on the expression of microtubule components in R-NSCLC tumor samples. Although the functional specificity of the various tubulin isotypes remains controversial, the expression patterns of certain tubulin isotypes are tissue-specific and the expression levels are correlated with the sensitivity to antitubulin agents (8–12). As access to frozen lung biopsy tissues for RNA analysis is often difficult, demonstrating that immunohistochemistry is able to provide useful predictive factors in NSCLC patients is likely to be beneficial for guiding the choice of chemotherapy regimens.

To assess whether the tubulin isotype level, as examined by immunohistochemistry, was able to identify predictive factors in R-NSCLC patients undergoing vinorelbine-based adjuvant chemotherapy, a retrospective study was performed on tumor samples from patients with R-NSCLC who were subsequently treated with vinorelbine-based regimens at The First Affiliated Hospital of Guangzhou Medical College (Guangzhou, China) between 2003 and 2008. The correlation between the biological results and the patient outcome was then investigated.

## Patients and methods

### Patient data

Tumor samples from 85 R-NSCLC patients treated between September 2003 and March 2008 at the First Affiliated Hospital of Guangzhou Medical College were analyzed. The histopathological subtypes were determined using the WHO classification for lung cancer. The current International Staging System for lung cancer was used for clinical disease staging (13). The clinicopathological characteristics of the patient population are shown in Table I. The median age at diagnosis was 57 years (range, 28–76 years). Subsequent to surgery, all patients were treated with vinorelbine in combination with cisplatin (CDDP) or carboplatin. The median follow-up time for the 85 patients was 31 months (range, 1–122 months), measured from the onset of chemotherapy.

The study was approved by the Ethics Committee of The First Affiliated Hospital of Guangzhou Medical College, Guangzhou, China. Written informed consent was obtained from the patients or patient’s family.

### Immunohistochemistry

The NSCLC tumor tissues were obtained by surgical excision. When possible, the diagnostic tumor blocks were used to ensure the availability of a sufficient number of viable, morphologically intact tumor cells to fulfill the scoring requirements and enable histopathological representation of the entire tumor. The tissue specimens were fixed in neutral buffered formalin, then embedded in paraffin wax and prepared as serial 3-*μ*m sections and attached to glass slides. The slides were placed in 10 mM citrate buffer (pH 6.0) for antigen retrieval, then the sections were incubated with normal rabbit serum, followed by anti-class II, III or IV β-tubulin antibodies (Sigma-Aldrich, St. Louis, MO, USA) at a dilution of 1:50. For the negative controls, the antibodies were substituted with phosphate-buffered saline (PBS).

Immunohistochemical staining was semi-quantitatively scored as −, +, ++ or +++ (score 3) on the basis of the percentage of positive cells (score 1) and the staining intensity (score 2), as shown in Table II. All slides were examined and scored independently by two pathologists blinded to the patient data.

### Chemotherapy

All patients received platinum-based regimens of 25 mg/m^2^ vinorelbine on days 1 and 8 and 75 mg/m^2^ CDDP on day 1 of a 21-day cycle (73 patients), or 25 mg/m^2^ vinorelbine on days 1 and 8 with carboplatin (dose equal to an area under the curve of 5; according to the Calvert formula) on day 1 of a 21-day cycle (12 patients). All patients received at least two courses of chemotherapy (range, 2–6 cycles).

### Follow-up and statistical analysis

The dates of recurrence and metastasis and any survival information were obtained from medical records, outpatient follow-up and telephone calls. Outcome data, including disease-free survival (DFS) and overall survival (OS), were calculated from the time of surgery to the date of tumor progression or mortality, respectively, or the last follow-up. The associations between immunohistochemical staining and the patient or tumor characteristics were examined using the χ^2^ test or Fisher’s exact test, as appropriate. Survival curves were estimated using the Kaplan-Meier method, while survival differences were compared with the log-rank test. The Cox proportional hazards model was used for multivariate analysis to assess the independent predictive value of β-tubulin expression. All tests were two-sided and P<0.05 was considered to a indicate statistically significant difference. All statistical analyses were performed using SPSS 13.0 (SPSS Inc., Chicago, IL, USA).

## Results

### Frequency of β-tubulin isotype expression in NSCLC

**Figure 1.**
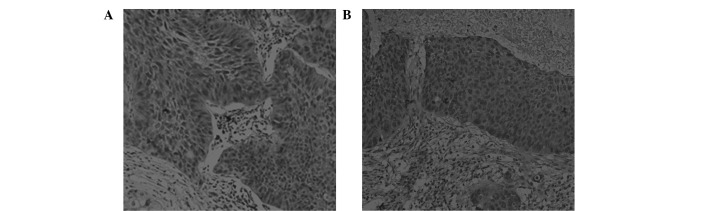
Adenocarcinoma of the lung stained with (A) anti-class II β-tubulin antibody and (B) anti-class III β-tubulin antibody. Magnification, ×200.

**Figure 2.**
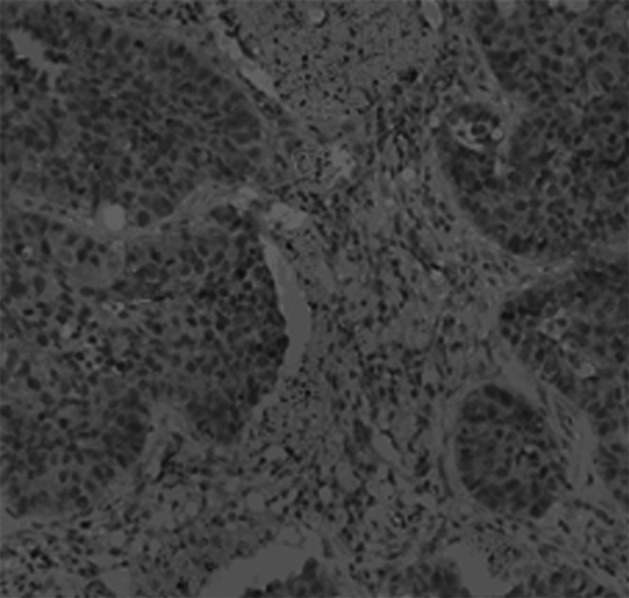
Adenocarcinoma of the lung stained with anti-class IV β-tubulin antibody. Magnification, ×200.

In the negative control slides, where the antibodies were substituted with PBS, no β-tubulin isotype expression was observed. Positive class III β-tubulin staining was observed in the cytoplasm of 78/85 (91.8%) of the tumor samples, while class II and class IV β-tubulin were positively expressed in 18/85 (21.2%) and 42/85 (49.4%) of the tumor samples, respectively. Representative examples of the β-tubulin immunohistochemical staining are shown in Figs. 1 and 2.

### Association of β-tubulin isotype expression and clinico-pathological features in NSCLC

The 85 patients were grouped according the tumor expression levels of the three β-tubulin isotypes. The patients were divided into high class III (score 3 of ++ or +++) and low class III β-tubulin expression (score 3 of − or +) based on the median class III β-tubulin expression score. As >50% of the patients did not express class II or class IV β-tubulin (78.8 and 50.6%, respectively), the patients were divided into negative and positive class II/IV β-tubulin groups. The tumor immunohistochemistry results are shown in Table III. Gender, age, histological type, smoking status, lymphatic metastasis status and tumor stage were not significantly associated with β-tubulin class II, III or IV expression (Table IV).

### Association of β-tubulin isotype expression with survival in NSCLC

**Figure 3.**
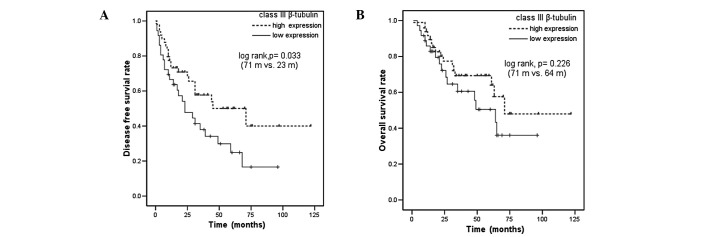
(A) DFS and (B) OS curves for R-NSCLC patients who received adjuvant chemotherapy, according to class III β-tubulin expression. (A) DFS was inferior in the class III β-tubulin-low patients. DFS, disease-free survival; OS, overall survival; R-NSCLC, resected non-small cell lung cancer.

**Figure 4.**
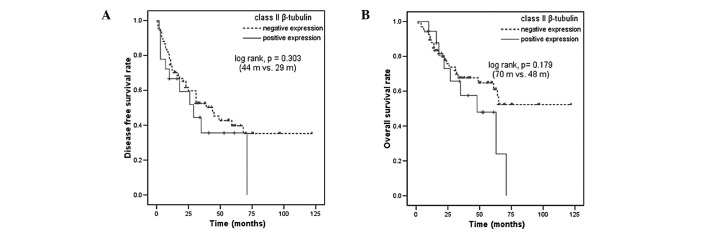
(A) DFS and (B) OS curves for R-NSCLC patients who received adjuvant chemotherapy, according to class II β-tubulin expression. DFS, disease-free survival; OS, overall survival; R-NSCLC, resected non-small cell lung cancer.

**Figure 5.**
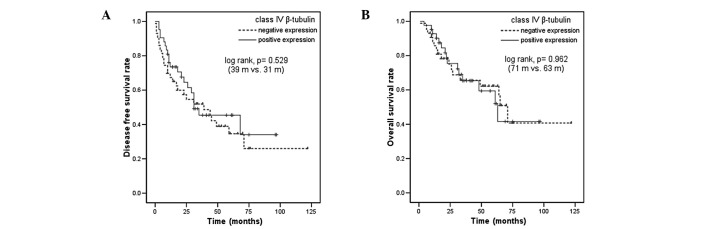
(A) DFS and (B) OS curves for R-NSCLC patients who recieved adjuvant chemotherapy, according to class IV β-tubulin expression. DFS, disease-free survival; OS, overall survival; R-NSCLC, resected non-small cell lung cancer.

High expression levels of class III β-tubulin were associated with longer median DFS compared with low class III β-tubulin expression (71 vs. 23 months, P=0.033; Fig. 3A). High expression levels of class III β-tubulin were also associated with a trend towards improved OS (71 vs. 64 months in patients with low level class III β-tubulin expression, P=0.226; Fig. 3B). DFS and OS were not significantly different in patients with positive or negative expression of class II or class IV β-tubulin (Figs. 4 and 5). Among the other patient characteristics, stage IV disease was associated with a shorter DFS (P=0.020) and shorter OS (P=0.005; data not shown).

### Multivariate analysis for DFS and OS

Multivariate analysis using the Cox proportional hazards model was performed to determine the independent predictive value of the β-tubulin isotypes. The multivariate analysis included the factors of gender, age, histology, stage and the expression of class II, III and IV β-tubulin and indicated that the disease stage and high class III β-tubulin expression were significant independent predictive factors for improved DFS (P=0.004 and P=0.031, respectively). High expression levels of class III β-tubulin yielded a hazard ratio of 0.61, with a 95% confidence interval ranging between 0.294 and 0.944. The tumor stage was correlated independently with OS (P=0.006), while gender, age, histology and class II, III or IV β-tubulin showed no correlation.

## Discussion

Since class II, III and IV β-tubulins have been reported to be associated with resistance to tubulin-binding agents (9–11), the frequency of β-tubulin isotype expression was investigated in R-NSCLC treated with vinorelbine-based adjuvant chemotherapy. The protein expression levels of class II, III and IV β-tubulin were investigated by immunohistochemistry and showed that class III > class IV > class II β-tubulin, with respect to relative abundance in R-NSCLC tumors. The expression levels of class II β-tubulin in the advanced tumors in a study by Sève *et al* appeared to be higher than those in R-NSCLC tumors in the present study (57% for ≥26% positive cells vs. 21.2% for ≥10% positive cells). The expression levels of class III β-tubulin appeared to be similiar between the advanced and resected NSCLC samples (14). To the best of our knowledge, class IV β-tubulin levels have not been previously reported in NSCLC tumors. No association was identified between the expression levels of the β-tubulin isotypes tested and the clinicopathological characteristics of R-NSCLC.

The present study is the first to show an association between the expression levels of microtubule proteins in R-NSCLC tumor samples and the patient outcome following vinorelbine-based adjuvant chemotherapy. The results of the present study suggested that high tumor expression levels of class III β-tubulin are associated with improved DFS and a trend towards longer OS following vinorelbine-based adjuvant chemotherapy in NSCLC patients. These observations are consistent with a previous study by Sève *et al* (2), which demonstrated that the expression levels of class III β-tubulin were correlated with DFS in NSCLC following vinorelbine-based adjuvant chemotherapy. Sève *et al* used semiquantitative immunohistochemistry to analyze the expression of class III β-tubulin in primary NSCLC tumor tissues obtained from 265/482 patients following treatment with vinorelbine/CDDP. It was observed that high levels of β-tubulin III expression were associated with a greater benefit from adjuvant chemo-therapy. These results contrasted with to the correlation between class III β-tubulin expression and chemotherapy in the advanced disease state (14). Consequently, this correlation remains controversial. The discrepancy between the predictive value of class III β-tubulin in the metastatic disease and the adjuvant setting is not without precedent. In colorectal cancer, the ability of thymidylate synthase to predict the benefit of chemotherapy differs in operable and advanced disease (15–17), and at present, the reasons for this difference remain unexplained.

Sève *et al* did not observe a correlation between the expression levels of class II or IV β-tubulin and DFS or OS following vinorelbine-based adjuvant chemotherapy, while in the present study, class II and IV β-tubulins were also not predictive of DFS or OS following vinorelbine-based adjuvant chemotherapy (2).

Hirai *et al* (18) used an *in vitro* histoculture drug response assay to measure the median effective dose (ED_50_) for vinorelbine. This showed that cells from R-NSCLC tumors expressing high levels of class III β-tubulin exhibited greater chemosensitivity to vinorelbine compared with cells from tumors with low levels of class III β-tubulin expression. This supports the present observation that class III β-tubulin has predictive value in NSCLC patients receiving vinorelbine-based adjuvant chemotherapy.

In conclusion, high levels of class III β-tubulin expression, as assessed by immunohistochemistry, were associated with an increased benefit from adjuvant vinorelbine-based chemo-therapy in patients with operable NSCLC. However, these results are not definitive and further study is required to confirm these observations and determine whether a class III β-tubulin immunohistochemical assay should be developed for use in clinics. Additionally, prospective randomized chemotherapy trials are required to further investigate the predictive value of class III β-tubulin expression, while preclinical investigations are required to clarify the mechanism by which β-tubulin affects the sensitivity of NSCLC cells to chemotherapy.

## Figures and Tables

**Table I.** Clinicopathological characteristics of the patient population.

Characteristic	No. of patients	DFS (months)	P-value
Total	85		
Gender			
Male	44	31.6	
Female	41	30.6	0.87
Age (years)			
≤60	50	28.9	
>60	38	34.1	0.37
Histology			
Adenocarcinoma	48	31.5	
Squamous cell carcinoma	29	28.7	
Other	8	37.3	0.70
Smoking			
Yes	36	33.2	
No	49	29.6	0.53
Lymphatic metastasis			
Yes	46	31.4	
No	39	30.7	0.91
Stage			
IB	10	47.4	
IIA	25	43.2	
IIB	33	21.9	
IIIA	17	21.6	0.006

DFS, disease-free survival.

**Table II.** Immunohistochemical scores.

Score 1		Score 2		Score 3	
		
Score	Positive cells (%)	Score	Intensity of staining	Score	Score 1 + score 2
0	<10	1	Weak	−	≤1
1	10–25	2	Moderate	+	2–3
2	25–50	3	Strong	++	4–5
3	50–75			+++	≥6
4	>75				

**Table III.** Results of immunostaining for class III, II and IV β-tubulin.

β-tubulin	Low expression (%)	High expression (%)	Negative expression (%)	Positive expression (%)
Class III	36 (42.4)	49 (57.6)	-	-
Class II	-	-	67 (78.8)	18 (21.2)
Class IV	-	-	43 (50.6)	42 (49.4)

**Table IV.** Comparison of baseline factors of the 85 patients stratified according to tubulin expression (types II, III and IV β-tubulin).

Factor	P-value	β-tubulin III expression (negative)		β-tubulin III expression (positive)		P-value	β-tubulin II expression (negative)		β-tubulin II expression (positive)		P-value	β-tubulin IV expression (negative)		β-tubulin IV expression (positive)	
					
		n	%	n	%		n	%	n	%		n	%	n	%
Gender	0.473					0.717					0.748				
Male		17	38.6	27	61.4		34	77.3	10	22.7		23	52.3	21	47.7
Female		19	46.3	22	53.7		33	80.5	8	19.5		20	48.8	21	51.2
Age (years)	0.600					0.446					0.897				
≤60		20	40.0	30	60.0		38	76.0	12	24.0		25	50.0	25	50.0
>60		16	45.7	19	54.3		29	82.9	6	17.1		18	51.4	17	48.6
Histology	0.724					0.962					0.308				
Adenocarcinoma		19	39.6	29	60.4		38	79.2	10	20.8		22	45.8	26	54.2
Squamous cell carcinoma		14	48.3	15	51.7		23	19.3	6	20.7		15	51.7	14	48.3
Other		3	37.5	5	62.5		6	75.0	2	25.0		6	75.0	2	25.0
Smoking	0.318					0.840					0.729				
No		23	46.9	26	53.1		39	79.6	10	20.4		24	49.0	25	51.0
Yes		13	36.1	23	63.9		28	77.8	8	22.2		19	52.8	17	47.2
Lymphatic metastasis	0.514					0.890					0.323				
Yes		18	39.1	28	60.9		36	78.3	10	21.7		21	46.7	24	53.3
No		18	46.2	21	53.8		31	79.5	8	21.2		21	55.3	17	44.7
Stage	0.603					0.652					0.336				
IB		5	50.0	5	50.0		9	90.0	1	10.0		4	40.0	6	60.0
IIA		8	32.0	17	68.0		20	80.0	5	20.0		13	52.0	12	48.0
IIB		16	48.5	17	51.5		24	72.7	9	27.3		20	60.6	13	39.4
IIIA		7	41.2	10	58.8		14	82.4	3	17.6		6	35.3	11	64.7

## References

[1] Jemal A, Bray F, Center MM (2011). Global cancer statistics. CA Cancer J Clin.

[2] Sève P, Lai R, Ding K (2007). Class III beta-tubulin expression and benefit from adjuvant cisplatin/vinorelbine chemotherapy in operable non-small cell lung cancer: analysis of NCIC JBR.10. Clin Cancer Res.

[3] Kato H, Ichinose Y, Ohta M (2004). A randomized trial of adjuvant chemotherapy with uracil-tegafur for adenocarcinoma of the lung. N Engl J Med.

[4] Arriagada R, Bergman B, Dunant A, International Adjuvant Lung Cancer Trial Collaborative Group (2004). Cisplatin-based adjuvant chemotherapy in patients with completely resected non-small-cell lung cancer. N Engl J Med.

[5] Hotta K, Matsuo K, Ueoka H (2004). Role of adjuvant chemotherapy in patients with resected non-small-cell lung cancer: reappraisal with a meta-analysis of randomized controlled trials. J Clin Oncol.

[6] Dumontet C, Sikic BI (1999). Mechanisms of action of and resistance to antitubulin agents: microtubule dynamics, drug transport, and cell death. J Clin Oncol.

[7] Sullivan KF (1988). Structure and utilization of tubulin isotypes. Annu Rev Cell Biol.

[8] Dumontet C, Durán GE, Steger KA, Murphy GL, Sussman HH, Sikic BI (1996). Differential expression of tubulin isotypes during the cell cycle. Cell Motil Cytoskeleton.

[9] Verdier-Pinard P, Wang F, Martello L, Burd B, Orr GA, Horwitz SB (2003). Analysis of tubulin isotypes and mutations from taxol-resistant cells by combined isoelectrofocusing and mass spectrometry. Biochemistry.

[10] Nicoletti MI, Valoti G, Giannakakou P (2001). Expression of beta-tubulin isotypes in human ovarian carcinoma xeno-grafts and in a sub-panel of human cancer cell lines from the NCI-Anticancer Drug Screen: correlation with sensitivity to microtubule active agents. Clin Cancer Res.

[11] Kavallaris M, Kuo DY, Burkhart CA (1997). Taxol resistant epithelial ovarian tumors are associated with altered expression of specific beta-tubulin isotypes. J Clin Invest.

[12] Ludueña RF (1993). Are tubulin isotypes functionally significant. Mol Biol Cell.

[13] Mountain CF (1997). Revisions in the International System for Staging Lung Cancer. Chest.

[14] Sève P, Isaac S, Trédan O (2005). Expression of class III (beta)-tubulin is predictive of patient outcome in patients with non-small cell lung cancer receiving vinorelbine-based chemotherapy. Clin Cancer Res.

[15] Aschele C, Debernardis D, Casazza S (1999). Immunohistochemical quantitation of thymidylate synthase expression in colorectal cancer metastases predicts for clinical outcome to fluorouracil-based chemotherapy. J Clin Oncol.

[16] Edler D, Glimelius B, Hallström M (2002). Thymidylate synthase expression in colorectal cancer: a prognostic and predictive marker of benefit from adjuvant fluorouracil-based chemotherapy. J Clin Oncol.

[17] Sarries C, Haura EB, Roig B (2002). Pharmacogenomic strategies for developing customized chemotherapy in non-small cell lung cancer. Pharmacogenomics.

[18] Hirai Y, Yoshimasu T, Oura S (2011). Is class III beta-tubulin a true predictive marker of sensitivity to vinorelbine in non-small cell lung cancer?. Chemosensitivity data evidence Anticancer Res.

